# A Human-Governed Clinical Informatics Framework for Safe AI-Assisted Mental Health Counseling: Secondary Framework Development and Requirement Mapping Study

**DOI:** 10.2196/103345

**Published:** 2026-08-21

**Authors:** Mi-Ae Yang, Kang-Su Ha

**Affiliations:** 1 Department of Science and Technology Convergence Graduate School Chosun University Gwangju Republic of Korea; 2 Department of Psychiatry Rainbow Hospital Gwangju Republic of Korea; 3 College of Medicine Chosun University Gwangju Republic of Korea

**Keywords:** clinical informatics, artificial intelligence, AI, mental health, counseling, natural language processing, human-governed workflow, decision support, data governance, patient safety, implementation science

## Abstract

**Background:**

Natural language processing and large language model systems are increasingly used to support mental health documentation, screening, and follow-up planning. In counseling contexts, model outputs may influence diagnostic framing, risk recognition, and clinical record content. Static performance metrics and fluent generated summaries are not sufficient to support safe implementation without governance, safety gating, human review, and monitoring.

**Objective:**

This study aimed to develop a human-governed clinical informatics framework for safe AI-assisted mental health counseling and make the formative evidence base and requirement-mapping process traceable.

**Methods:**

We conducted a secondary framework development and requirement mapping study using the Korean AI Hub psychological counseling dataset, official data description and use documents, released KLUE-BERT risk prediction model materials, released KoAlpaca summary generation resources, and a deidentified 139-case rule-based summary safety screening audit table derived from the original summary comparison file. Raw counseling transcript text, reference summary full text, and generated summary full text are not included in the manuscript or supplementary materials. We extracted failure modes from documented data and model characteristics, released code and configuration files, documentation-reported model metrics, and rule-based proxy flags. Each failure mode was mapped to safety controls, operational criteria, and deployment-level requirements.

**Results:**

The official documents described 1661 counseling sessions and 465,474 paragraph-level tokens across depression, anxiety disorder, addiction, and normal control groups. Of the 1661 sessions, the documented split included 1339 (80.6%) training, 173 (10.4%) validation, and 149 (9%) test sessions. The summary generation materials documented 1278 training summaries and 139 test summaries. Documentation-reported model metrics included KLUE-BERT accuracies of 71.43% for depression, 73.53% for anxiety, and 66.67% for addiction and KoAlpaca BERTScore precision, recall, and *F*_1_-score values of 62.13%, 59.56%, and 60.80%, respectively. The 139-case screening table contained 77 (55.4%) depression, 31 (22.3%) anxiety, and 31 (22.3%) addiction cases. Rule trigger rates included unsupported content proxy flags in 41% (57/139) of cases, overdiagnostic expression proxy flags in 31.7% (44/139) of cases, medicalized expression proxy flags in 54.7% (76/139) of cases, and any rule-based proxy flag in 91.4% (127/139) of cases. These values are conservative rule trigger rates rather than confirmed clinical error rates. The findings informed a 7-stage workflow, 6 safety control layers, an operational safety gate, a workflow-to-control crosswalk, deployment-level transition criteria, and a constructed high-risk example.

**Conclusions:**

AI-assisted mental health counseling should be implemented as a governed clinical information workflow rather than as an autonomous diagnostic or documentation pathway. The proposed framework specifies safeguards and validation requirements for future supervised evaluations, but it does not itself establish clinical safety or clinical effectiveness. Prospective simulation, clinician usability testing, patient or client feedback, and independent expert validation remain necessary before routine deployment.

## Introduction

Mental health services increasingly rely on digital tools for documentation, triage, screening, and follow-up [1,2]. Counseling encounters contain longitudinal information about mood, anxiety, addiction, interpersonal stress, trauma exposure, self-harm, functional impairment, and protective factors [3]. Natural language processing and large language model technologies may help summarize counseling encounters and detect risk-related signals, but they also create safety challenges when outputs are presented as clinically meaningful information [1-4].

Counseling AI is not merely a text processing problem. A model-generated risk label or summary can affect how clinicians understand a client, how a record is written, how follow-up decisions are framed, and how risk is escalated [1,2]. False-positive risk predictions may create unnecessary diagnostic labeling and alert fatigue, whereas false-negative outputs or summaries that omit risk signals may reduce recognition of self-harm, violence, addiction, trauma-related danger, or severe functional deterioration [5-9]. Fluent but unsupported summaries may appear clinically authoritative even when they contain hallucinated, overdiagnostic, or poorly calibrated content [3,7,10].

Clinical AI implementation requires more than model checkpoints, demonstration data, or accuracy scores [3-7]. It requires a sociotechnical workflow that defines who reviews AI output, when output may influence documentation, how risk signals are escalated, how errors are reported, and how model behavior is monitored after deployment [5,10,11]. For mental health AI, these governance requirements are especially important because language-based outputs may influence stigma, safety planning, diagnostic framing, and trust in care [3,8-11].

This study aimed to develop a traceable, human-governed clinical informatics framework for safe AI-assisted mental health counseling. The framework is intended for supervised research and pilot settings in hospitals, counseling centers, digital mental health services, and research teams that evaluate or pilot Korean-language AI-assisted counseling resources. The term “human-governed” is used to describe the intended implementation structure: AI output remains advisory and requires documented human review before it affects records or decisions. The framework was not developed through a prospective human-in-the-loop clinical trial.

## Methods

### Study Design

We conducted a secondary framework development and requirement mapping study. This study did not develop a new autonomous AI model, prospectively test clinical effectiveness, or use a prospective human-in-the-loop component during framework development. Instead, it translated documented data and model characteristics and rule-triggered summary-screening signals from a Korean-language counseling AI use case into a clinical informatics framework. The study design followed design science logic: problem identification, evidence source selection, failure mode extraction, requirement mapping, artifact construction, and specification of future validation needs.

### Data Source and AI Hub Documentation

The development context was the publicly released AI Hub psychological counseling dataset [12], its official data description [13], and its use guideline [14]. The official documents described 1661 counseling sessions and 465,474 paragraph-level tokens across depression, anxiety disorder, addiction, and normal control groups. The data structure included transcript text and JSON labeling files. The AI Hub data description states that deidentified MP3 audio and transcribed TXT files were produced during data construction, but MP3 audio files were not publicly released because of privacy protection rules; only TXT files were made available as public source data [13]. Of the 1661 sessions, the official guideline documented training, validation, and test splits of 1339 (80.6%), 173 (10.4%), and 149 (9%) sessions, respectively. For the summary generation task, 1278 training summaries and 139 test summaries were documented; normal control cases were excluded from summary generation modeling when symptom, risk, and improvement factors were not applicable.

The annotation schema contained session-level metadata, diagnostic group labels, summary text, silence and total counseling time, paragraph-level speaker and utterance information, symptom factors, risk factors, symptom change factors, and intervention factors. Examples of safety-relevant fields included “suicidal,” “trauma_experience,” “sleep_disturbance,” “irritability,” “craving,” “withdrawal,” “emotional_regulation,” “social_support,” and “cognitive_restructuring.” These fields were used to characterize the counseling AI use case and define failure modes that could affect risk recognition or documentation quality.

### Reference Model Materials

Released reference model documentation included KLUE-BERT–based risk prediction materials and KoAlpaca 4-bit summary generation materials [15]. The KoAlpaca summary generation resources were treated as released AI Hub reference model materials documented in the same AI Hub reference model package [15]. The KLUE-BERT risk prediction model was based on bidirectional encoder representations from transformers (BERT) and Korean Language Understanding Evaluation (KLUE)–related resources [16,17]. The reference model documentation described the KLUE-BERT model as receiving a speaker-marked counseling transcript string and producing a 0 or 1 prediction for depression, anxiety, or addiction risk. The released model folders confirmed disease-specific trained model directories for depression, anxiety, and addiction, each containing model configuration, tokenizer configuration, special token mapping, vocabulary, a training argument file, and model weights in safetensors format. In the inspected depression model configuration, the base model was klue/bert-base, the architecture was CustomBertForSequenceRegression, max_position_embeddings was 512, and tokenizer_config specified a model_max_length of 512.

The inspected KLUE-BERT code did not provide probability-calibrated raw scores, area under the receiver operating characteristic curve, sensitivity, specificity, or confusion matrix export files. The inference logic was implemented as a regression-style scalar output followed by rounding to an integer in the range of 0 to 3 and conversion to a binary zero-vs-nonzero indicator. Therefore, KLUE-BERT model materials were treated as documentation and code-level traceability inputs rather than independent performance reproduction evidence.

The summary generation model used KoAlpaca 4-bit resources based on EleutherAI/polyglot-ko-12.8b. The inspected model package included training and inference scripts, low-rank adaptation adapter configuration, tokenizer files, and model configuration files. The low-rank adaptation configuration specified *r* of 8, alpha of 32, dropout of 0.05, and query_key_value as the target module. The inference script used the instruction prompt “다음과 같은 상담기록을 보고 요약서를 작성해주세요” “Please review the following counseling record and write a summary.” and generated summaries from the original text field in bertscore_evaluation.xlsx. The released materials were distributed as Colab-executable notebooks and Python source code resources rather than Docker-based container images.

### Rule-Based Summary Safety Screening Audit Source and Deidentification

For summary screening traceability, we used a 139-case summary comparison file containing case file names, original counseling transcripts, reference summaries, and generated summaries. In this manuscript, “audit” means a retrospective automated formative screening procedure applied to summary characteristics; it does not mean an independent clinical safety audit or psychiatrist-adjudicated outcome review. Because raw counseling text is sensitive, the original transcript text, reference summary full text, and generated summary full text were removed before reporting. A deidentified rule-based summary safety screening table was created with anonymized case identifiers, diagnostic group labels, summary length indicators, section presence indicators, and rule-based proxy flags. This file is provided as Multimedia Appendix 1. The proxy flags were used to support traceability of aggregate screening signals and were not treated as independent clinical expert judgments.

### Proxy Rule Development and Reproducibility

The proxy rules were implemented by the technical author and reviewed by the clinical coauthor. The clinical coauthor reviewed the operational gate domains, keyword categories, failure mode mapping, and worked example to ensure that the rules were clinically interpretable as conservative screening prompts rather than confirmed clinical errors. The initial screening domains were specified before computing the final aggregate counts by using the AI Hub schema fields, expected summary sections, and known counseling safety concerns. After preliminary inspection, category names were clarified for reporting consistency, but the final rule definitions and aggregate counts were computed from the locked rule set described in Multimedia Appendix 1.

The screening procedure compared section presence and keyword presence between reference summary text and generated summary text before those texts were removed from the reporting file. The main rule families were (1) required section omission rules for risk, improvement, and intervention sections; (2) high-risk keyword omission rules for self-harm and suicide, trauma and violence, and addiction risk categories; (3) unsupported content proxy rules for critical clinical or risk-related keywords that appeared in generated summaries without support in the original transcript or reference summary; (4) overdiagnostic or medicalized expression rules; and (5) length ratio rules. Generated-to-reference length ratios below 0.50 were classified as too short, and ratios above 1.50 were classified as too long. These thresholds were chosen as conservative heuristics to identify unusually compressed or expanded summaries requiring human review, not as validated clinical thresholds. No formal manual false positive or false negative adjudication study was conducted; this limitation is stated explicitly.

### Failure Mode Identification and Control Mapping

We identified failure modes from four sources: (1) official data and model documentation; (2) released code, model folders, and execution resource files; (3) documentation-reported model results and the absence of raw score or confusion matrix exports; and (4) rule-based proxy flags from the 139-case screening table. Failure modes were grouped into data and labeling, model, output, human review, privacy, and governance categories. Recognized bias categories from clinical AI literature, including label bias, class imbalance, calibration uncertainty, missing external validation, data leakage concerns, automation bias, and deployment drift, were used as a cross-check so that the failure mode list would not rely solely on author judgment [8,9].

Each failure mode was mapped to a minimum required control using the following rule: if a failure mode could alter risk recognition, diagnostic framing, record content, privacy protection, or accountability, then the framework required a control that prevented automatic use of the AI output, required a documented human decision, or triggered monitoring or governance review. In the workflow-to-control crosswalk, “Required” means that the control is a minimum condition for that workflow stage because it prevents uncontrolled record integration, risk omission, privacy leakage, or accountability gaps. “Support” means that the control reinforces safety but is not the minimum gatekeeping condition for that specific stage.

### Safety Gate and Deployment-Level Criteria

The safety gate was operationalized as a prerecord integration checkpoint. A generated summary or risk flag may proceed to clinician review only if it has passed source consistency screening, high risk omission screening, overdiagnostic language screening, privacy screening, and required section screening. In routine deployment, source consistency should be assessed against the original counseling source or other clinically available evidence because reference summaries may not be available outside retrospective evaluation. If any gate item is positive, the output remains a draft and must be edited, rejected, escalated, or accompanied by additional assessment before final record integration. High-risk content related to self-harm, suicide, violence, abuse, severe functional deterioration, withdrawal, intoxication, or relapse cannot be silently dismissed; the reviewer must document the rationale for the final decision.

Deployment levels were defined as transition states rather than descriptive labels. Progression from one level to another requires documented evidence of local safety, review burden, error handling, and governance sign-off. Level 0 is offline research only. Level 1 permits supervised internal pilot-testing with mandatory clinician review. Level 2 permits limited decision support pilot use after local validation and escalation compliance review. Level 3 permits routine supervised use only if postdeployment monitoring demonstrates acceptable safety, workflow burden, and incident response. Level 4 autonomous use is not recommended for AI-assisted mental health counseling under the current evidence base.

### Ethical Considerations

No participants were recruited, no intervention was delivered, and the authors did not contact human participants for the present secondary framework development analysis. The study used released AI Hub documentation, released reference model resources, and an author-generated deidentified rule-based screening table derived from the 139-case summary comparison resource. Raw counseling transcripts, reference summary full text, generated summary full text, and identifiable excerpts were not reproduced in the manuscript or supplementary materials.

The official AI Hub use guideline for psychological counseling data describes the original data construction process, participant explanation and consent procedures, privacy safeguards, and deidentification procedures for transcript and audio data [14]. According to the guideline, textual identifiers were replaced with entity markers, such as “@NAME,” “@ADDRESS,” “@PHONE,” “@DOB,” “@EMAIL,” “@SCHOOL,” and “@HOSPITAL,” and audio data were deidentified through voice transformation, masking, silence insertion, or beep processing [14]. The guideline also indicates that consent for personal information use was obtained during the original data construction [14].

Because this secondary data analysis used anonymized and publicly accessible data, it was exempt from approval by the institutional review board under local regulatory policies. The ethical rationale was considered in relation to general human subject research principles [18]; the Korean Bioethics and Safety Act definition and review context for human subject research [19]; and the consent, privacy, confidentiality, intrusiveness, and potential harm considerations discussed by Eysenbach and Till [20] for sensitive online or community research. Before any prospective implementation, local deployment, or patient- or client-facing use, institutional ethics, privacy, and governance review would be required according to local policy.

## Results

### Evidence Inventory and Traceability

The framework was derived from a traceable set of documentation, model resource, and deidentified screening inputs. These inputs did not establish prospective clinical safety, but they identified where controls were needed before any counseling AI output could be used in documentation, triage, or follow-up planning. Table 1 summarizes how each evidence source contributed to a safety signal or framework requirement.

**Table 1:** Evidence sources and traceability for framework development.

Evidence source	Traceable item	Derived signal or design implication	Framework use
AI Hub data description and use guideline	1661 sessions; 465,474 paragraph-level tokens; depression, anxiety, addiction, and normal groups; JSON schema	Counseling data contain risk, symptom, change, and intervention fields that can influence risk recognition and documentation	Data governance, annotation audit, and safety gate requirements
Official training, validation, and test documentation	1339 training, 173 validation, and 149 test sessions; summary task with 1278 training and 139 test summaries	Model resources are tied to a defined but nonclinical validation structure	Deployment levels require local validation before clinical use
KLUE-BERT released code and model folders	Disease-specific folders for depression, anxiety, and addiction; configuration and tokenizer files; model_max_length=512 in inspected depression model	Model identity and execution structure are traceable, but raw score, AUROC^a^, specificity, sensitivity, and confusion matrix exports were not available	Model governance, version lock, threshold policy, and limitation statement
KoAlpaca code and summary generation files	LoRA^b^ adapter, inference script, prompt, generation settings, and bertscore_evaluation.xlsx input	Generated summaries can be screened for omitted risk information, unsupported content, and unsafe wording	Output layer safety gate and mandatory human review
139-case deidentified rule-based screening table	77 depression cases, 31 anxiety cases, and 31 addiction cases; full transcript text removed	Rule-based proxy flags identify outputs requiring structured review; flags are not expert clinical judgments	Traceable aggregate screening signals and worked example

^a^AUROC: area under the receiver operating characteristic curve.

^b^LoRA: low-rank adaptation.

### Dataset and Reference Model Characteristics

The AI Hub resources documented a balanced clinical use case across depression, anxiety disorder, addiction, and normal control groups. The normal control group was smaller than each clinical group, and the released classification resources reported accuracy values rather than full clinical validation statistics. For this reason, the framework treats model results as decision support signals that require local validation before operational use. Table 2 summarizes the dataset and reference model characteristics.

**Table 2:** Dataset and reference model characteristics.

Domain	Documentation-confirmed characteristic	Safety implication
Dataset composition	Depression: 484 sessions; anxiety: 487 sessions; addiction: 448 sessions; normal control: 242 sessions	Local deployment should examine class distribution and alert burden
Annotation schema	Session-level labels, paragraph text, symptom_factor, risk_factor, symptom_change, and intervention_factor	Label validity and paragraph-level scoring should be audited before treating risk fields as clinical truth
Risk prediction model	KLUE-BERT model for 0 or 1 prediction of depression, anxiety, or addiction	Outputs should be interpreted as decision support signals, not diagnoses
Summary model	KoAlpaca 4-bit model for structured summary reports	Generated summaries require source consistency and omission screening
Reported classification performance	Official accuracies: 71.43% for depression, 73.53% for anxiety, 66.67% for addiction, and 70.54% weighted average	Documentation-reported accuracy alone is insufficient for clinical deployment; sensitivity, specificity, calibration, and subgroup behavior remain required
Reported summary performance	Official BERTScore precision: 62.13%; recall: 59.56%; *F*_1_-score: 60.80%	Semantic similarity does not establish clinical safety; omission and unsupported content checks remain required

### Rule-Based Proxy Criteria

The proxy criteria were designed to identify outputs that should be reviewed by a human, not label outputs as confirmed clinical errors. Table 3 summarizes the main rule families and the interpretation of positive flags. Positive flags may reflect conservative screening rules and do not necessarily indicate clinically harmful outputs.

**Table 3:** Rule-based proxy criteria and interpretation.

Rule family	Operational rule	Interpretation
Section omission	Expected reference summary section is present, but the corresponding generated summary section is absent	Possible completeness issue requiring structured review
High-risk keyword omission	Reference or source content contains self-harm or suicide, trauma or violence, or addiction risk keywords not reflected in the generated summary	Possible high risk omission requiring clinician review
Unsupported content proxy	Generated critical clinical or risk keywords are absent from both the reference summary and original transcript	Possible unsupported content requiring source consistency review
Overdiagnostic or medicalized expression proxy	Generated summary contains diagnostic certainty; disease, treatment, or patient language; or related medicalized terms	Possible terminology or framing issue requiring editing
Length ratio rule	Generated-to-reference length ratio of <0.50 or >1.50	Unusually short or long summary requiring completeness and review burden assessment

### The 139-Case Rule-Based Screening Findings

The deidentified 139-case screening table retained only anonymized identifiers, diagnostic group labels, summary length indicators, section presence indicators, and rule-based proxy flags. It did not retain raw transcript text, reference summary text, or generated summary text. The group distribution was 55.4% (n=77) depression cases, 22.3% (n=31) anxiety cases, and 22.3% (n=31) addiction cases.

Rule trigger rates showed that generated summaries frequently required structured review under conservative screening rules. Section-level omissions were less frequent than language or content proxy flags: risk factor section omission occurred in 2.9% (4/139) of cases, improvement factor omission occurred in 8.6% (12/139) of cases, and intervention factor omission occurred in 13.7% (19/139) of cases. High risk omission proxy flags were observed for self-harm and suicide keywords in 5.8% (8/139) of cases, trauma and violence keywords in 15.1% (21/139) of cases, and addiction risk keywords in 13.7% (19/139) of cases. Unsupported content proxy flags occurred in 41% (57/139) of cases, overdiagnostic expression proxy flags occurred in 31.7% (44/139) of cases, and medicalized expression proxy flags occurred in 54.7% (76/139) of cases. These results show why human review and source consistency checking are needed, but they should not be interpreted as confirmed hallucination rates, clinical error rates, or unsafe summary rates. Table 4 summarizes the aggregate rule-based summary safety-screening proxy flags by diagnostic group.

**Table 4:** Aggregate rule-based summary safety screening proxy flags. Positive flags reflect conservative automated rule triggers and do not necessarily indicate clinically harmful outputs or independently adjudicated clinical errors.

Rule-based proxy flag	Overall count, n/N (%)	Depression, n/N (%)	Anxiety, n/N (%)	Addiction, n/N (%)	Interpretation for framework design
Risk factor section omission	4/139 (2.9)	2/4 (50)	1/4 (25)	1/4 (25)	Generated summaries require a structured risk factor section check before record integration
Improvement factor section omission	12/139 (8.6)	4/12 (33.3)	4/12 (33.3)	4/12 (33.3)	Generated summaries may omit recovery or protective content relevant to follow-up planning
Intervention factor section omission	19/139 (13.7)	7/19 (36.8)	5/19 (26.3)	7/19 (36.8)	Counselor intervention content should be checked before summaries are used for continuity of care
Self-harm or suicide keyword omission	8/139 (5.8)	5/8 (62.5)	2/8 (25)	1/8 (12.5)	Possible omission requires mandatory human review and escalation logic
Trauma or violence keyword omission	21/139 (15.1)	15/21 (71.4)	6/21 (28.6)	0/21 (0)	Trauma- or violence-related content requires high-risk checklist screening
Addiction risk keyword omission	19/139 (13.7)	10/19 (52.6)	6/19 (31.6)	3/19 (15.8)	Addiction-related risk content requires relapse, withdrawal, or intoxication screening
Unsupported content proxy	57/139 (41)	34/57 (59.6)	15/57 (26.3)	8/57 (14)	Fluent generated text requires source consistency review before it is trusted
Overdiagnostic expression proxy	44/139 (31.7)	27/44 (61.4)	9/44 (20.5)	8/44 (18.2)	Diagnostic certainty and stigmatizing language require terminology guardrails
Medicalized expression proxy	76/139 (54.7)	42/76 (55.3)	15/76 (19.7)	19/76 (25)	Medicalized wording should be reviewed to avoid inappropriate clinical framing
Generated summary too short	34/139 (24.5)	15/34 (44.1)	8/34 (23.5)	11/34 (32.4)	Abnormally short summaries require completeness review
Generated summary too long	5/139 (3.6)	3/5 (60)	1/5 (20)	1/5 (20)	Overly long summaries may increase review burden and require editing
Any rule-based proxy flag	127/139 (91.4)	75/127 (59.1)	25/127 (19.7)	27/127 (21.3)	Most draft outputs triggered at least one conservative review rule

### Failure Modes and Required Controls

The identified failure modes show how a documentation-level AI resource can create implementation risks if outputs are treated as clinically authoritative. The corresponding controls are designed to keep generated summaries and risk outputs in a draft or decision support state until a responsible human reviewer has assessed them. Table 5 summarizes the failure mode–to-control mapping.

**Table 5:** Failure modes and required controls.

Failure mode	Evidence source	Potential harm	Required control	Minimum operational criterion
Label and annotation uncertainty	Official schema and paragraph-level scoring; nonexpert and expert review documented, but independent reliability not available	Risk labels may inherit annotator assumptions or class distribution effects	Label audit control	Before pilot use, document class distribution, missingness, and interrater or expert review process for local labels
Positive or poorly calibrated risk prediction	Official accuracy only; no raw score, sensitivity, specificity, calibration, or confusion matrix exports available	Unnecessary labeling, alert fatigue, and missed false negative analysis	Model governance control	Local validation must report sensitivity, specificity, calibration, false positive rate, false negative rate, and review burden before level 2 use
Omitted high-risk information	139-case rule trigger proxy flags	Self-harm, trauma, violence, addiction, or deterioration may be missed	Safety gate checklist	Any high-risk keyword or missing risk section triggers mandatory clinician review and documented disposition
Unsupported or hallucinated summary content	Unsupported content proxy and official hallucination prevention documentation	Unsupported content may enter clinical record	Source consistency control	Reviewer must compare AI draft against the original source or clinically available source evidence before approval
Overdiagnostic or medicalized wording	Overdiagnostic and medicalized expression proxy flags	Stigma, premature diagnosis, and inappropriate referral or treatment framing	Terminology guardrail	AI output must avoid definitive diagnosis unless confirmed by a qualified clinician; uncertain language must be edited
Automation bias	Known clinical AI risk and framework use case	Clinicians may overtrust fluent text	Interface and human review control	Output displayed as draft; final record requires active human confirmation, edit, or rejection
Privacy leakage	AI Hub deidentification rules and data use restrictions	Sensitive counseling information may be disclosed or redistributed	Data governance control	Raw transcripts not redistributed; role-based access and deidentification retained
Accountability gap	Need for record integration and incident response	Unclear responsibility after AI-related error	Governance control	Named clinical owner, audit trail, incident pathway, and model version record required

### Proposed Human-Governed Framework

**Figure 1:**
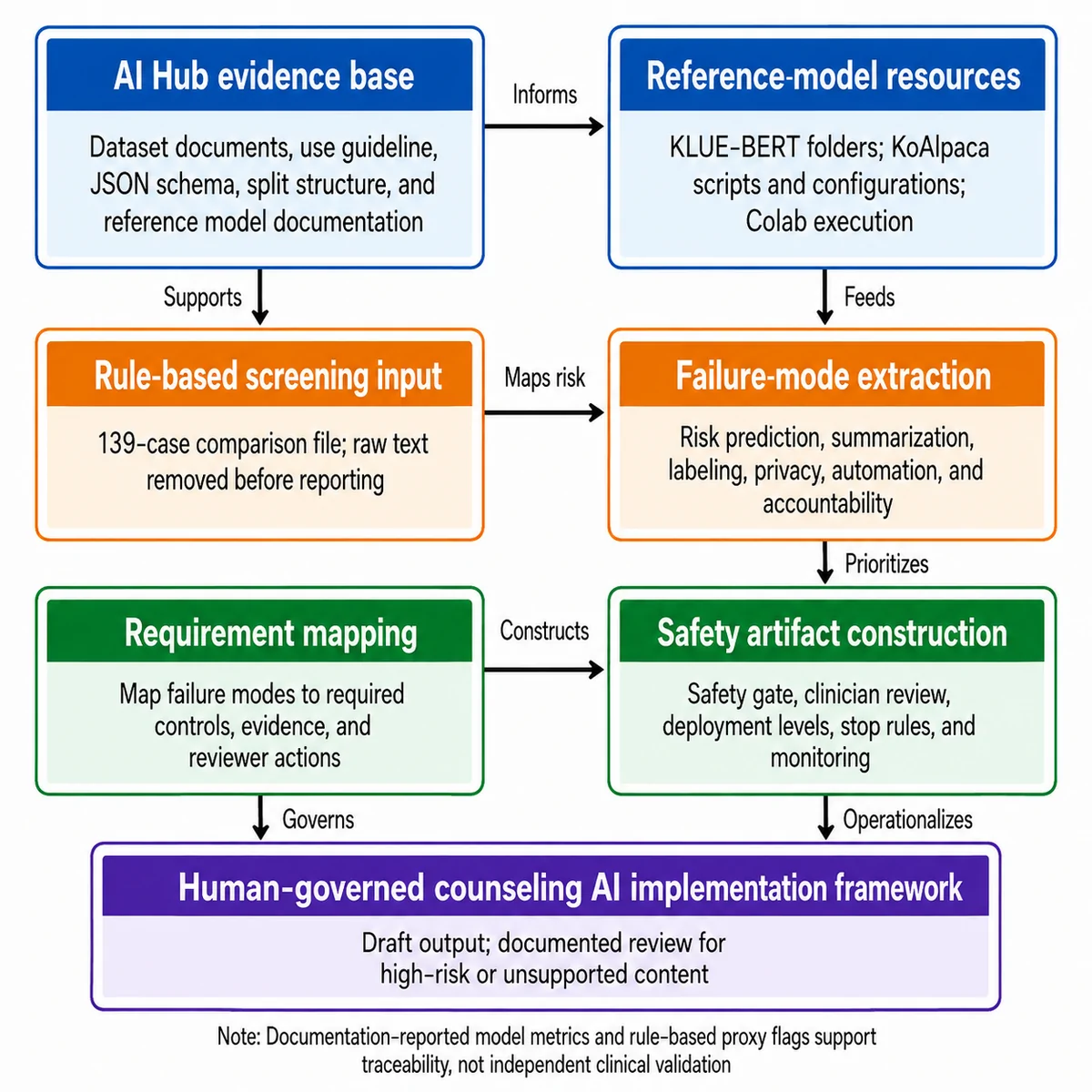
Human-governed framework development workflow. The figure shows how AI Hub documentation, model resources, and the 139-case deidentified rule-based summary safety screening audit were converted into failure modes, requirements, and a human-governed counseling AI implementation framework.

The framework consists of seven workflow stages: (1) data intake and deidentification, (2) AI service execution, (3) structured safety gating, (4) clinician review, (5) final record integration, (6) postdeployment monitoring, and (7) institutional governance. These stages are paired with 6 control layers: governance, data, model, output, human review, and postdeployment surveillance. AI output is always treated as draft or decision support content. It is not a final diagnosis, final counseling note, or autonomous escalation decision. The framework development workflow is shown in Figure 1.

**Figure 2:**
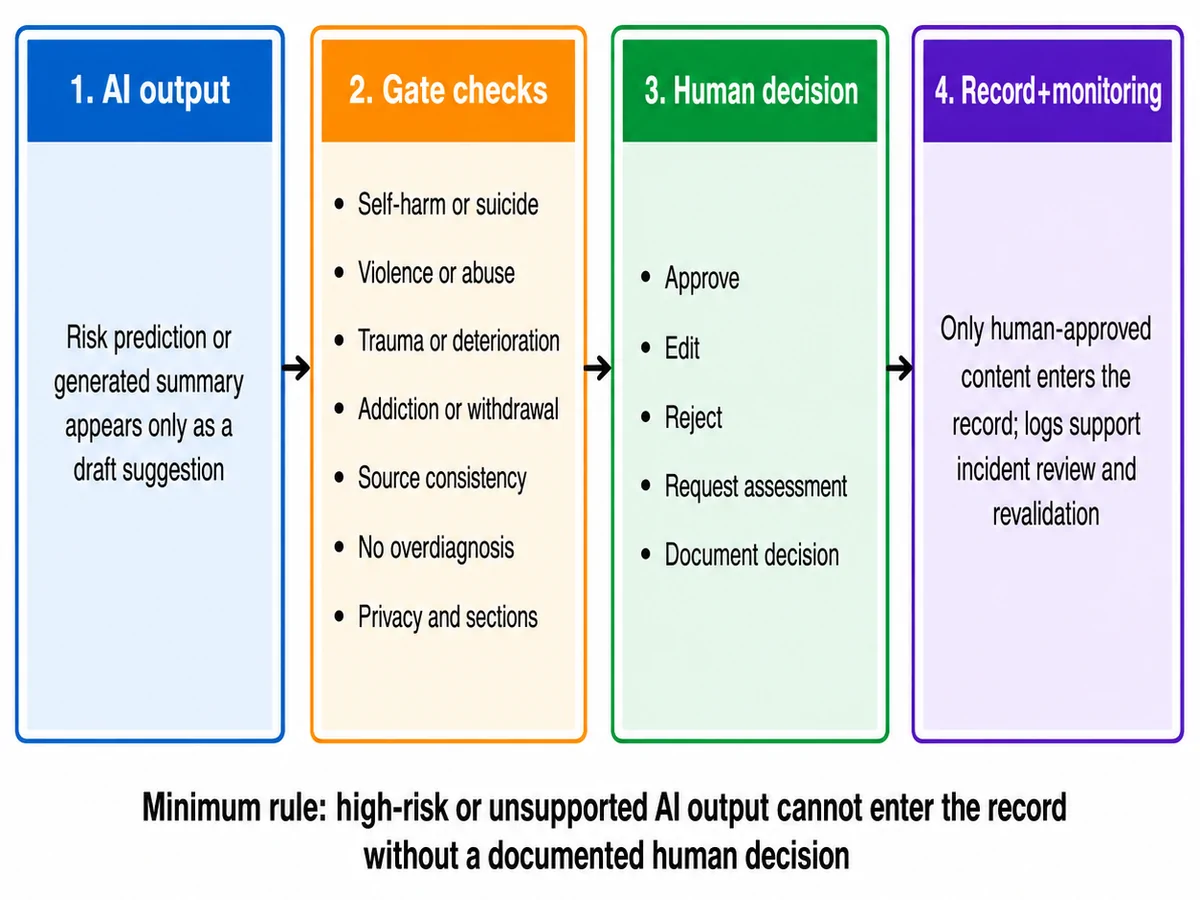
Operational safety gate for AI-assisted counseling output. AI-generated outputs remain as draft suggestions until source consistency, high risk omission, overdiagnostic wording, privacy, and required section checks have been completed and a human decision has been documented.

Figure 2 operationalizes the framework as a 4-step prerecord safety pathway. AI-generated risk predictions or summaries initially remain as draft suggestions and are screened for high-risk omissions, unsupported content, overdiagnostic wording, privacy concerns, and required section completeness. A clinician must then approve, edit, reject, or request additional assessment, and only human-approved content may proceed to record integration and postdeployment monitoring.

**Figure 3:**
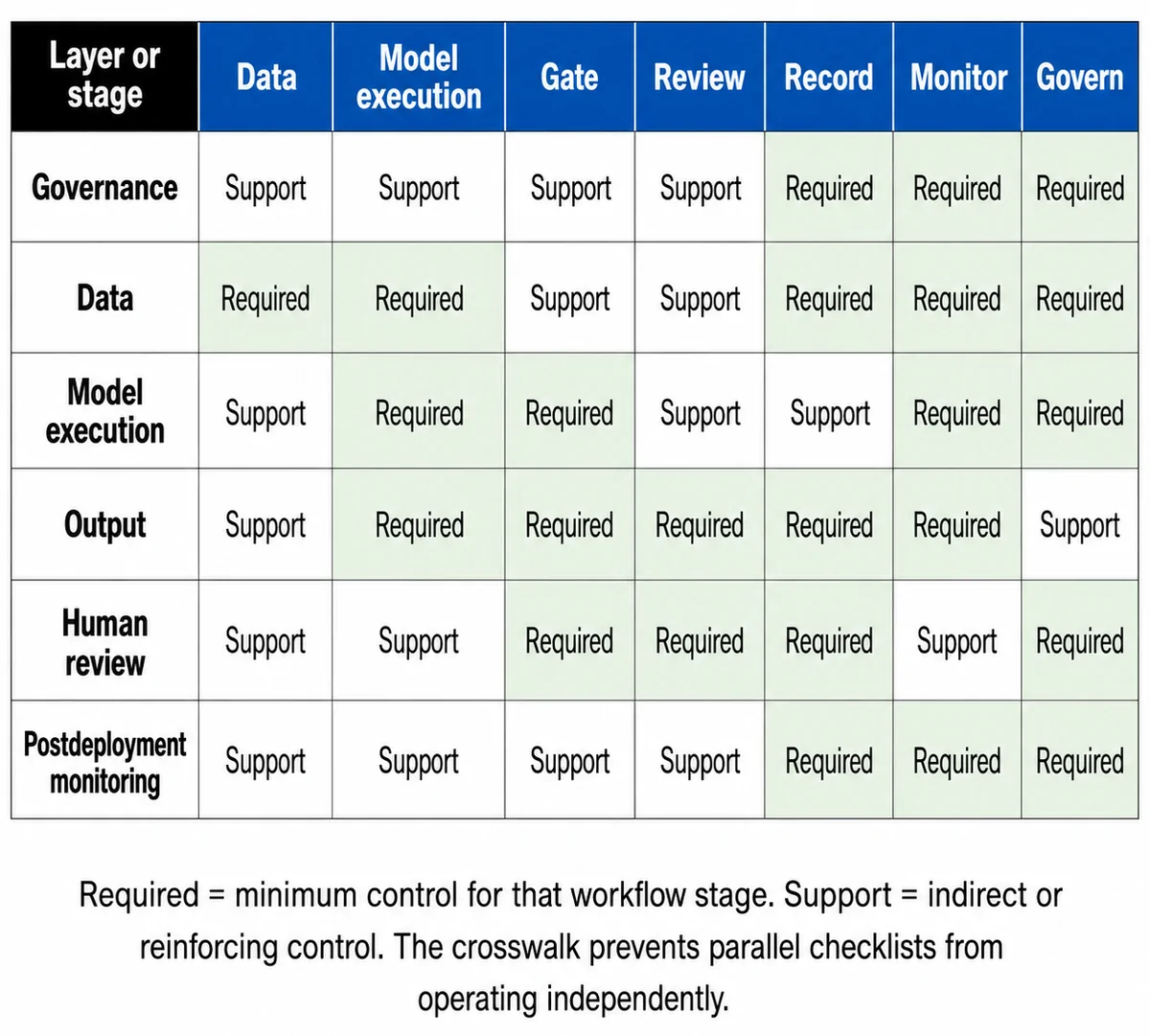
Workflow stage–to–control layer crosswalk. “Required” indicates a minimum control for that workflow stage because it prevents uncontrolled record integration, risk omission, privacy leakage, or accountability gaps. “Support” indicates an indirect or reinforcing control. The crosswalk links workflow stages to safety control layers so that governance, data, model execution, output, human review, and postdeployment controls are not treated as independent checklists.

Figure 3 shows how the 6 safety control layers are distributed across the 7 workflow stages rather than applied as isolated checklists. Required cells identify the minimum gatekeeping control for each stage, whereas support cells indicate indirect or reinforcing controls. The crosswalk clarifies where governance, data, model execution, output, human review, and postdeployment responsibilities become mandatory throughout the implementation pathway.

### Operational Safety Gate

The operational safety gate domains, pass criteria, trigger criteria, and required actions are summarized in Table 6. The gate is intentionally conservative: a positive proxy flag should lead to structured review, not automatic rejection or automatic clinical escalation. Human reviewers remain responsible for final interpretation and for applying local crisis or clinical protocols.

**Table 6:** Operational safety gate criteria.

Gate domain	Pass criterion	Fail or trigger criterion	Required action
Source consistency	Generated summary claims can be supported by the original source or other clinically available evidence	New diagnostic claims, unsupported facts, or inconsistent content appear	Edit or reject AI output; document reason
Self-harm or suicide	No self-harm or suicide signal is present, or any signal is accurately represented	Source content suggests self-harm or suicide, but the generated output omits or minimizes it	Immediate clinician review; escalation according to local crisis protocol
Violence, abuse, or trauma	Relevant content is accurately represented when present	Trauma, violence, abuse, or severe threat content is omitted or softened	Clinician review and risk documentation required
Addiction risk	Relapse, withdrawal, intoxication, craving, or loss of control is represented when present	Addiction risk content is omitted, minimized, or reframed incorrectly	Addiction risk review; assess need for follow-up or escalation
Overdiagnosis or medicalization	Output uses cautious, descriptive language	Output states or implies a diagnosis without clinician confirmation or uses stigmatizing certainty	Terminology editing or rejection before record integration
Privacy	No identifiers or sensitive redistributable text appear	Identifier or raw transcript text appears in output or logs beyond approved use	Remove content, report incident if required, and block record integration
Completeness	Required sections are present when applicable: symptoms, risk factors, improvement factors, and intervention factors	Missing required section or abnormal summary length	Manual completeness review before approval

### Deployment-Level Transition Criteria

The deployment-level transition criteria and authorization requirements are summarized in Table 7. The levels are intended to prevent premature clinical use. Higher levels require local validation, escalation compliance, monitoring, and governance sign-off; autonomous use is not authorized under the current evidence base.

**Table 7:** Deployment-level transition criteria.

Level	Permitted use	Required evidence before entering level	Authorization and stop rule
Level 0: offline research only	Retrospective analysis, sandbox testing, and nonclinical framework development	Data use permission; no patient- or client-facing output; no record integration	Study lead authorization; stop if raw identifiers or restricted data are exposed
Level 1: supervised internal pilot	Clinician-reviewed draft summaries or safety check support in a controlled internal setting	Model version locked; safety gate configured; reviewer training completed; audit logging enabled	Clinical owner and governance lead sign-off; stop if high risk omissions or unsupported content recur
Level 2: limited decision support pilot	Restricted triage or documentation support with mandatory clinician confirmation	Local validation reports sensitivity, specificity, false positive and false negative rates, calibration, summary review burden, and escalation compliance	Institutional AI or clinical governance sign-off; suspend if safety gate failures exceed local tolerance or escalation compliance is incomplete
Level 3: routine supervised use	Routine supervised documentation or decision support use with monitoring	Postdeployment monitoring shows stable performance, acceptable review burden, error resolution process, user training, and periodic revalidation	Formal institutional approval; stop if drift, unsafe output pattern, or incident review indicates unacceptable risk
Level 4: autonomous use	AI output directly affects records or decisions without human confirmation	Not recommended for AI-assisted mental health counseling under current evidence	Not authorized

### Constructed Worked Example: Possible Self-Harm Risk Omission

The following worked example is a constructed illustrative example based on a generalized high-risk pattern, not a direct quotation from any of the 139 cases and not a modified identifiable case. A counseling source contains indirect references to hopelessness, inability to continue, and family burden. The AI-generated summary describes anxiety and sleep disturbance but does not mention self-harm–related concern or hopelessness. Under the proposed framework, the safety gate identifies a self-harm or suicide omission proxy because clinically available source evidence contains high-risk language not reflected in the generated summary. The system prevents automatic record integration, displays the output as a draft, and requires clinician review. The clinician reviews the source evidence, edits the summary to include a nonstigmatizing risk statement when appropriate, documents a brief rationale, and follows local crisis escalation protocols if immediate risk is suspected. The final record stores only the human-approved summary, the safety gate flag, the reviewer identity, the decision time stamp, and the follow-up action. The case is also logged for postdeployment monitoring of recurring omission patterns.

## Discussion

### Principal Findings

This study developed a human-governed clinical informatics framework for AI-assisted mental health counseling. The main finding is that counseling AI should be governed as a clinical information system rather than judged only by model metrics. By making the evidence base traceable, the study showed how AI Hub documentation, released model materials, and a 139-case deidentified rule-based summary safety screening table can be converted into workflow controls, safety gate criteria, deployment levels, and monitoring requirements.

### Contribution Beyond Existing AI Governance Guidance

The framework is consistent with general health AI governance, clinical prediction reporting, implementation guidance, and clinical decision support literature [3-7,10,11,21,22]. Its specific contribution is the counseling-focused operationalization of those principles. First, it treats generated counseling summaries as draft clinical information that must be checked for risk omission, overdiagnostic wording, and unsupported content before record integration. Second, it includes a safety gate tailored to mental health risks such as self-harm, trauma, violence, addiction, and severe functional decline. Third, it links model governance to workflow decisions by requiring local sensitivity, specificity, calibration, error burden, and escalation compliance evidence before higher deployment levels. The framework also reflects the need to address algorithmic bias and distributional effects before AI outputs influence care pathways [23]. Fourth, it specifies that autonomous use is not recommended for AI-assisted mental health counseling under the current evidence base.

### Implications for Hospitals and Counseling Centers

Institutions considering AI-assisted mental health counseling should begin with level 0 or level 1 use. In practice, this means offline research or supervised internal pilots in which AI-generated summaries and risk predictions are clearly labeled as drafts. Staff should be trained to recognize automation bias and apply the high-risk checklist consistently. Before any system is used for routine triage or documentation support, local leaders should define thresholds, escalation rules, audit responsibilities, privacy procedures, user training, and stopping rules.

In supervised pilot settings, institutions should disclose to patients or clients when AI-generated draft summaries or risk support tools are used in documentation, triage, or the counseling workflow while clarifying that final interpretation and decisions remain the responsibility of a qualified human professional. At minimum, AI disclosure should be required when AI output influences the clinical record, triage workflow, or client-facing communication.

### Implications for Developers

Developers should design counseling AI interfaces around clinician control rather than maximum automation. Useful features include provenance metadata, model version display, source-linked summary review, high-risk checklist prompts, edit history, reject and escalation options, and dashboards for error monitoring. Risk scores or generated summaries should not be presented as diagnostic conclusions unless they have been clinically validated for that specific purpose and setting.

Model layer governance should also consider interpretability and model complexity. When 2 systems provide comparable local safety performance, the more interpretable or easier-to-audit system should be preferred for mental health counseling workflows because it reduces the verification burden placed on human reviewers and supports accountability.

### Limitations

This study has several limitations. First, it was a secondary framework development study, not a prospective implementation trial. Second, no clinician usability study, patient or client feedback study, expert Delphi panel, or independent external validation was conducted. Third, the framework was developed from a Korean-language AI Hub counseling use case and may require adaptation for other languages, populations, or service settings. Fourth, KLUE-BERT raw prediction scores, area under the receiver operating characteristic curve, sensitivity, specificity, calibration curves, and confusion matrix exports were not available in the released materials reviewed; therefore, the manuscript reports documentation-level model characteristics rather than independently reproduced risk prediction performance. Fifth, the rule-based proxy flags were automated screening triggers intended for traceability and structured review; they were not psychiatrist-adjudicated clinical safety outcomes and should not be interpreted as clinical error or unsafe output rates. Sixth, no formal manual false positive or false negative adjudication study was performed for the proxy flags. Seventh, participant compensation details from the original AI Hub data collection were not available in the released documents reviewed. Eighth, local legal, ethical, and institutional requirements may differ and should be reviewed before any deployment.

### Future Research

Future studies should prospectively evaluate the framework in simulated and real counseling workflows. Priority outcomes include clinician review time, high-risk information recall, false alert burden, documentation quality, summary correction rate, escalation compliance, incident frequency, calibration drift, patient or client acceptability, and clinician workload. Future work should incorporate independent expert review of rule-based proxy flags and local validation of risk prediction models using sensitivity, specificity, calibration, subgroup performance, and decision curve analysis.

### Conclusions

AI-assisted mental health counseling should be implemented through clinician-governed workflows rather than autonomous documentation or diagnostic pathways. The proposed framework connects data governance, model control, operational safety gating, clinician review, record integration, monitoring, and institutional accountability. The framework specifies safeguards and validation requirements for future supervised evaluation, but it does not itself establish clinical safety or clinical effectiveness.

## Data Availability

The underlying psychological counseling dataset and reference model resources are available through the AI Hub subject to its data use procedures and restrictions. The authors cannot redistribute raw counseling transcripts, original labeling files, audio files, or original AI Hub data because they may contain sensitive mental health information and are subject to AI Hub data use conditions. A deidentified, author-generated rule-based summary safety screening table that does not contain raw counseling text, reference summary text, and generated summary text is provided as Multimedia Appendix 1.

## Appendix

Multimedia Appendix 1 Deidentified 139-case rule-based summary safety screening flag table derived from bertscore_evaluation.xlsx. Raw counseling transcript text, reference summary text, and generated summary text were removed.

## Abbreviations

BERT: bidirectional encoder representations from transformers
KLUE: Korean Language Understanding Evaluation
